# METTL3-Mediated lncSNHG7 m^6^A Modification in the Osteogenic/Odontogenic Differentiation of Human Dental Stem Cells

**DOI:** 10.3390/jcm12010113

**Published:** 2022-12-23

**Authors:** Yeqing Yang, Junkai Zeng, Chong Jiang, Jiawen Chen, Ci Song, Ming Chen, Buling Wu

**Affiliations:** 1 Nanfang Hospital, Southern Medical University, 1838 Guangzhou Avenue North, Guangzhou 510515, China; 2 Stomatological Hospital, Southern Medical University, No. 366 Jiangnan Avenue South, Haizhu District, Guangzhou 510280, China; 3 School of Stomatology, Southern Medical University, Guangzhou Avenue North, Guangzhou 510515, China; 4 Guangdong Provincial People’s Hospital, Guangdong Academy of Medical Sciences, Guangzhou 510080, China; 5 Shenzhen Stomatology Hospital (Pingshan), Southern Medical University, 143 Dongzong Road, Pingshan District, Shenzhen 518118, China

**Keywords:** human dental pulp stem cells, N6-methyladenosine, osteogenic/odontogenic differentiation, RNA epigenetics, long noncoding RNA

## Abstract

**Background**: Human dental pulp stem cells (hDPSCs) play an important role in endodontic regeneration. N6-methyladenosine (m^6^A) is the most common RNA modification, and noncoding RNAs have also been demonstrated to have regulatory roles in the expression of m^6^A regulatory proteins. However, the study on m^6^A modification in hDPSCs has not yet been conducted. **Methods**: Single base site PCR (MazF) was used to detect the m^6^A modification site of lncSNHG7 before and after mineralization of hDPSCs to screen the target m^6^A modification protein, and bioinformatics analysis was used to analyze the related pathways rich in lncSNHG7. After knockdown and overexpression of lncSNHG7 and METTL3, the osteogenic/odontogenic ability was detected. After METTL3 knockdown, the m^6^A modification level and its expression of lncSNHG7 were detected by MazF, and their binding was confirmed. Finally, the effects of lncSNHG7 and METTL3 on the Wnt/β-catenin pathway were detected. **Results**: MazF experiments revealed that lncSNHG7 had a m^6^A modification before and after mineralization of hDPSCs, and the occurrence site was 2081. METTL3 was most significantly upregulated after mineralization of hDPSCs. Knockdown/ overexpression of lncSNHG7 and METTL3 inhibited/promoted the osteogenic/odontogenic differentiation of hDPSCs. The m^6^A modification and expression of lncSNHG7 were both regulated by METTL3. Subsequently, lncSNHG7 and METTL3 were found to regulate the Wnt/β-catenin signaling pathway. **Conclusion**: These results revealed that METTL3 can activate the Wnt/β-catenin signaling pathway by regulating the m^6^A modification and expression of lncSNHG7 in hDPSCs to enhance the osteogenic/odontogenic differentiation of hDPSCs. Our study provides new insight into stem cell-based tissue engineering.

## 1. Introduction

Bone tissue engineering is based on the concepts of stem cells, growth factors and scaffold materials [1,2]. Many situations, such as trauma, tumor and necrosis, lead to bone defects, which may, eventually, lead to extensive dysfunction [3]. For example, if bacterial invasion and other factors exceed the resistance of the pulp tissue itself, it will further develop into pulpitis and periapical periodontitis [4]. Clinically regenerative endodontic treatment is considered the ideal treatment for necrotic permanent teeth [5]. Therefore, the introduction of effective new strategies to achieve functional and physiological bone reconstruction is urgently required to improve the potential of pulp tissue repair. Human dental pulp stem cells (hDPSCs), initially identified by Gronthos [6], have lower immunogenicity, and higher proliferation rates and cloning potential compared with mesenchymal stem cells [7,8]; moreover, hDPSCs from different species can form regenerative tissue after implantation in animals. In addition, hDPSCs come from a wide range of sources, in sufficient quantity and as a convenient material, showing great potential in regenerative medicine for the treatment of various human diseases; as a consequence, they present good potential for clinical transformation. However, the exact mechanism of osteogenic/odontogenic differentiation is still unclear and requires investigation to achieve the best clinical bone enhancement results.

N6 methyladenosine (m^6^A) is the most common modification method in mRNA and was implicated in all aspects of posttranscriptional RNA metabolism [9]. The widespread presence of m^6^A in the human transcriptome has aroused great interest among researchers. Exploration of methylation patterns in cells can not only reveal the specific distribution of the m^6^A modification in many transcripts, but also the differences in m^6^A status under different physiological conditions [10]. The biological function of m^6^A modification mainly depends on methyltransferases, demethylases and methylated reading proteins. Among them, methyltransferases such as methyltransferase 3 (METTL3) were most extensively studied: their main role is to catalyze the m^6^A modification of adenosine on RNA [11]. It was demonstrated that m^6^A modification plays an important role in cancer, metabolism, embryonic stem cell processes and tissue development [12,13,14]. Studies have shown that the m^6^A modification mediated by METTL3 can promote the osteogenic differentiation of bone marrow mesenchymal stem cells through different pathways and help to inhibit the progression of osteoporosis [15]. In addition, the m^6^A modification of METTL3 can also promote osteogenic differentiation in human adipose-derived stem cells induced by NEL-like 1 protein [16]. For hDPSCs, it was shown that the m^6^A modification of METTL3 has a regulatory role in the cell cycle [17] and METTL3 might affect the LPS-induced inflammatory response by regulating the alternative splicing of MyD88 [18]. However, research on the osteogenic/odontogenic differentiation of hDPSCs is still lacking; improving the osteogenic/odontogenic differentiation ability of hDPSCs is a key issue to be solved before their clinical application and transformation. Therefore, the effects and mechanisms of m^6^A modification on the osteogenic/odontogenic differentiation of hDPSCs require further exploration.

Many factors are involved in regulating osteogenic/odontogenic differentiation of mesenchymal stem cells. Among them, long noncoding RNAs (lncRNAs), as a large class of regulatory molecules, have attracted much attention in recent years. A type of noncoding RNA (ncRNA) with a length of more than 200 nucleotides, lncRNAs cannot be translated into protein. Studies have shown that lncRNAs are involved in a variety of biological processes and disease pathogenesis, and they play a significant role in the osteogenic/odontogenic differentiation of stem cells [19,20]. An increasing number of studies have also shown that lncRNAs can affect the osteogenic/odontogenic differentiation of hDPSCs by regulating the expression of downstream target genes in combination with microRNAs (miRNAs) [21,22,23]. However, current research on whether lncRNAs can play a regulatory role in this process is still in the preliminary stage, and the functions and mechanisms of a large number of lncRNAs are still unclear. In addition, because hDPSCs are ideal seed cells for regenerative tissue engineering, it is of clinical significance to improve their osteogenic/odontogenic differentiation ability through in vitro editing technology before implantation in vivo. Thus far, there has been no research on the regulation of lncRNA m^6^A modification in the process of hDPSC osteogenic/odontogenic differentiation, so further research is required.

In this study, and for the first time, m^6^A modification of lncRNAs was combined with the hDPSC osteogenic/odontogenic differentiation pathway: it confirmed the promoting effect of METTL3 in the osteogenic/odontogenic differentiation of hDPSCs; it also confirmed the regulatory effect of METTL3 on the m^6^A modification of lncRNA SNHG7 and its relationship with the Wnt/β-catenin signaling pathway. The aim was to provide a new concept and method for bone tissue engineering.

## 2. Methods

### 2.1. hDPSCs Culture and Characterization

The hDPSCs used in this study were obtained from healthy third molar teeth (without caries and intact after extraction) from 15 patients (6 males and 9 females) aged 18–25 years; the teeth were indicated for extraction at the Department of Stomatology in Nanfang Hospital, Southern Medical University, Guangzhou, Guangdong, China. All experimental protocols were approved by the Ethical Committee, Southern Medical University (NFEC-2022-173). As previously described in [24], the hDPSCs were cultured in Dulbecco’s modified Eagle’s medium (DMEM) with 10% fetal bovine serum (FBS; GIBCO, Life Technologies, NSW, Australia), 100 U/mL penicillin and 100 μg/mL streptomycin (Sigma, St. Louis, MO, USA) added, at a temperature of 37 °C, and air containing 5% CO_2_. The medium was changed every 3 days, and hDPSCs at passages 3–5 were used for the subsequent experiments [25,26]. We divided the samples into two groups: the undifferentiated hDPSC group, in which cells were cultured in 10% FBS in DMEM with no supplements, and the differentiated hDPSC group, in which cells were cultured in 50 mg/mL ascorbic acid, 100 nmol/l dexamethasone and 10 mmol/l β-glycerophosphate (Sigma, St Louis, MO, USA) in DMEM for 14 days. Flow cytometry was performed to identify the phenotypes of the hDPSCs by screening the surface markers against CD29, CD44, CD90, CD45 and CD34.

### 2.2. Single Base Site PCR (MazF)

The conserved motif region (m^6^A ACA site) of the core ACA sequence on lncSNHG7 was verified. The m^6^A modification levels in the hDPSC undifferentiated group, differentiated group and after METTL3 knockdown were detected. The RNA endonuclease MazF recognized the RNA single strand and cleaved at the 5′ end of the unmethylated ACA site but could not cleave the methylated m^6^A ACA site. The extracted total RNA samples were divided into two parts, one without MazF treatment and the other after MazF treatment. The m^6^A methylation level of specific ACA sites in the samples was then detected by real-time quantitative polymerase chain reaction (qRT-PCR) [27,28].

### 2.3. Alkaline Phosphatase (ALP) and Alizarin Red Staining (ARS)

Samples were first washed three times with phosphate-buffered saline and were fixed in 4% paraformaldehyde for 15 min. After washing, the hDPSCs were stained with the NBT/BCIP Staining Kit and Alizarin red. The results for each group were photographed under an inverted microscope.

### 2.4. Real-Time Polymerase Chain Reaction

The total RNAs were isolated from the undifferentiated hDPSC and differentiated hDPSC groups, obtained by culturing, as described above. A quantity of 1 μg of RNA per sample was reverse-transcribed into cDNA using a cDNA Reverse Transcription Kit (Takara, Tokyo, Japan). qRT-PCR was performed in a 20 μL reaction system. Finally, the relative expression of RNAs was calculated using the 2^−ΔΔCt^ method with glyceraldehyde-3-phosphate dehydrogenase (GAPDH) as the reference gene. Each sample was taken in triplicate, and the results were obtained from the independent experiments. The primer sequences used in the real-time PCR are summarized in Table 1.

### 2.5. Western Blot Analysis

The hDPSC protein was lysed by radioimmunoprecipitation assay buffer (Pierce, Rockford, IL, USA). The lysate containing loading buffer (2% SDS and 1% 2-mercaptoethanol) was prepared at 99 °C for 5 min. The samples were separated on 10% SDS polyacrylamide gel and transferred to 0.22 μm polyvinylidene fluoride membranes using a semidry transfer apparatus. Afterward, the membranes were blocked with skim milk powder solution at room temperature for 1 h. The transferred proteins were reacted with the primary antibody overnight at 4 °C and then labeled with the secondary antibody for 1 h at room temperature. Primary antibodies in this study included METTL3, GAPDH, dentin sialophosphoprotein (DSPP), dentin matrix acidic phosphoprotein 1 (DMP1), runt-related transcription factor 2 (Runx2) and ALP, phosphorylation-GSK-3β, and GSK-3β and β-catenin. Immunoreactive proteins were detected by using the ECL Kit (Beyotime Biotech, Shanghai, China), and the band densities were quantified using Image J software (National Institutes of Health, MD, USA) (v1.8.0).

### 2.6. Gene Knockdown and Overexpression

The undifferentiated hDPSC group and differentiated hDPSC group were cultured as described above and seeded into six-well plates at a density of 2 × 10^5^ cells per well. Transfection was performed when cells had grown to 60–80% confluency according to the instruction manual. For METTL3 and lncSNHG7 knockdown, the small interfering RNAs (siRNAs) for METTL3, lncSNHG7 and negative control siRNA were synthesized by Genechem (Shanghai, China). The overexpression lentivirus of METTL3 and lncSNHG7 were also synthesized by Genechem (Shanghai, China). The transfection procedure followed the manufacturer’s instructions.

### 2.7. Bioinformatic Analysis

The m^6^A modification position of lncSNHG7 was predicted by the SRAMP website tool. The binding site of lncSNHG7 to METTL3 was predicted using the catRAPID website, and the interaction probability of lncSNHG7 to METTL3 was predicted using the RPISeq website. Differentially expressed lncRNAs during the osteogenic differentiation of hDPSCs were analyzed using GEO2R in GSE138179 [29] and SRP214747 [30]. m^6^Avar, WHISTLE software was used to predict the ACA sites where m^6^A modification may occur in lncSNHG7. The Starbase database was used to predict related m^6^A-modifying enzymes that may bind to lncSNHG7. Both Gene Ontology (GO) analysis and analysis using the Kyoto Encyclopedia of Genes and Genomes (KEGG) were carried out. GO (http://geneontology.org/, Accessed on 21 September 2021) enrichment analysis was used to define gene attributes in organisms from three fields: biological processes (BP), cellular components (CC) and molecular functions (MF) (*p* < 0.05 was used). David software was used to test the statistical enrichment of the target gene candidates in the KEGG pathway database (KEGG; https://david.ncifcrf.gov/, Accessed on 21 September 2021).

### 2.8. RNA-Binding Protein Immunoprecipitation (RIP) Assay

Based on the manufacturer’s instructions, the RIP assay was removed with an RNA-Binding Protein Immunoprecipitation Kit. Cells were dissolved with RIP lysis buffer. Cell lysates (100 μL) were treated with the RIP buffer and cultured with Proteinase K and magnetic beads conjugated with anti-METTL3 antibody or control (anti-lgG) (Millipore, Burlington, MA, USA). The RNA bound to the beads was purified and then reverse-transcribed into cDNA for qRT-PCR.

### 2.9. Statistical Analysis

All experiments were performed three times. The data were processed by SPSS 25.0 software (SPSS, Chicago, IL, USA). Analysis of variance and Student’s *t*-test were used to evaluate statistical differences in different groups. All results were summarized and shown as means ± standard deviation. Results were treated with statistical significance at *p* < 0.05. One-way analysis of variance (ANOVA) followed by Dunnett’s post hoc test was used for multiple group comparisons. Prism software (Graphpad Prism Software, CA, USA) (v8.2.1.441) was used to create the figures.

## 3. Results

### 3.1. Characteristics of hDPSCs

**Figure 1:**
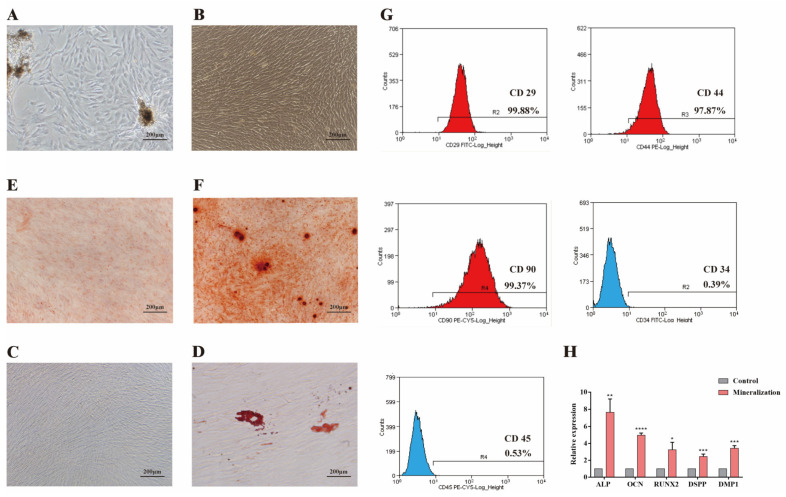
Culture and identification of hDPSCs. (**A**,**B**) Primary cultured and passage hDPSCs. (**C**,**D**) Oil red O staining of hDPSCs showed that no lipid droplet was observed in the undifferentiated hDPSCs group but a few lipid droplets were found in the differentiated hDPSCs group. (**E**,**F**) Alizarin Red S staining of hDPSCs showed that no obvious nodules in the undifferentiated hDPSCs but obviously more nodules and mineralized matrix were formed in the differentiated hDPSCs group. (**G**) The flow cytometric analysis revealed that hDPSCs were positive for mesenchymal stem cell marker (CD29, CD44, CD90) but were negative for hematopoietic stem cell marker (CD34, CD45). (**H**) mRNA expressions of osteogenic/odontogenic genes ALP, OCN, RUNX2, DSPP and DMP1 were detected by qRT-PCR. All samples were performed in triplicate. The data are represented as means ± SD. * *p* < 0.05, ** *p* < 0.01, *** *p* < 0.001, **** *p* < 0.0001.

hDPSCs were extracted from the third molars of healthy people. Primary cultured hDPSCs grew around the tissue blocks (Figure 1A). Morphological observation showed that the cells had a fibroblast-like appearance (Figure 1B). To further identify the multidirectional differentiation potential of hDPSCs, the isolated hDPSCs were induced to differentiate into osteoblasts and adipocytes. Lipid droplets were observed in the cytoplasm using Oil Red O staining, and the matrix mineralization increased significantly in the process of osteogenic induction compared with the undifferentiated group (Figure 1C–F). In addition, hDPSCs exhibited a high expression of CD29 (99.88%), CD44 (97.87%) and CD90 (99.37%), but were negative for CD34 (0.39%) and CD45 (0.53%) (Figure 1G). The qRT–PCR results suggest that the expression levels of DSPP, DMP1, RUNX2, ALP were upregulated (Figure 1H).

### 3.2. lncSNHG7 m^6^A Modification in hDPSCs

**Figure 2:**
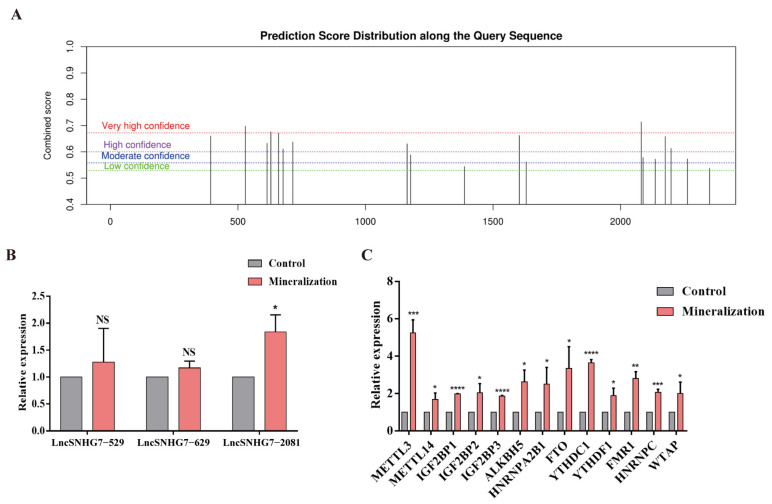
The m^6^A modification on lncSNHG7. (**A**) The m^6^A prediction score distribution of lncSNHG7 predicted by SRAMP website tools. (**B**) Single base site PCR (MazF) analysis was used to confirm the m^6^A modification site of lncSNHG7 after mineralization. (**C**) mRNA expressions of m^6^A modification-related enzymes detected by qRT-PCR. The data were represented as means ± SD for each group: * *p* < 0.05, ** *p* < 0.01, *** *p* < 0.001, **** *p* < 0.0001. NS: Not Statistically Significant.

By analyzing the GSE138179 and SRP214747 datasets, we found that the expression of lncSNHG7 was enhanced after osteogenic/odontogenic differentiation of hDPSCs. Through m^6^Avar, the WHISTLE database predicted that lncSNHG7 might have 19 m^6^A modification sites (Supplementary Table S1 and Figure 2A), among which there were three ACA modification sites with very high confidence. The m^6^A single base site PCR (MazF) verified that lncSNHG7 had an m^6^A modification on the 2081 ACA site (Figure 2B). Thus, according to the results of the StarBase database, m^6^A-related modifying enzymes that might bind to lncSNHG7 include METTL3/14, IGF2BP1/2/3, ALKBH5, HNRNPA2B1, FTO, YTHDC1, YTHDF1, FMR1, HNRNPC and WTAP (Supplementary Table S2). The expression of all m^6^A-related enzymes was detected in hDPSCs, and it was found that the expression levels of most of them increased in hDPSCs after osteogenic/odontogenic differentiation (*p* < 0.05). METTL3 exhibited the highest expression (Figure 2C).

### 3.3. METTL3 Promoted Osteogenic/Odontogenic Differentiation of hDPSCs

**Figure 3:**
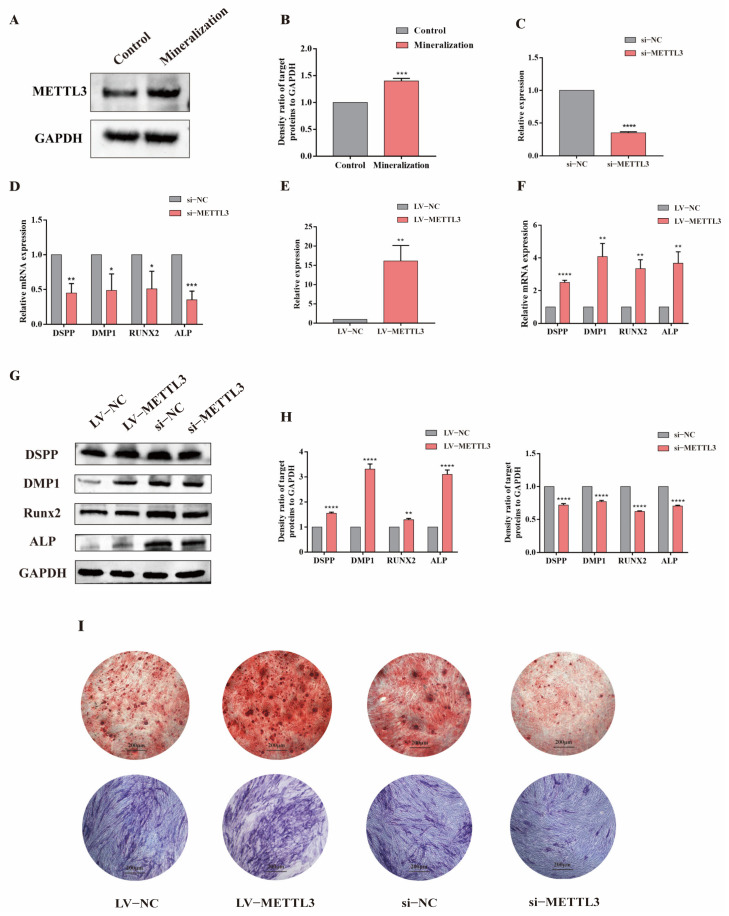
METTL3 promoted osteogenic/odontogenic differentiation of hDPSCs. (**A**) The protein expression of METTL3 detected by Western blot. (**B**) The density ratio of target proteins to GAPDH. (**C**,**E**) The knockdown and overexpression effect of si-METTL3 and LV-METTL3 detected by qRT-PCR. (**D**,**F**) mRNA expressions of osteogenic/odontogenic genes detected by qRT-PCR after the knockdown and overexpression of METTL3. (**G**) Western blot results showed the expression level of osteogenic/odontogenic proteins decreased in the si-METTL3 group and increased in the LV-METTL3 after mineralization. (**H**) The density ratio of target proteins to GAPDH. (**I**) ARS and ALP staining after the knockdown and overexpression of METTL3. The data were represented as means ± SD for each group: * *p* < 0.05, ** *p* < 0.01, *** *p* < 0.001, **** *p* < 0.0001.

After mineralization of the hDPSCs, the protein level of METTL3 increased (Figure 3A,B). We speculated that METTL3 might be involved in the regulation of the osteogenic/odontogenic differentiation of hDPSCs. We then knocked down METTL3 using siRNA and the efficiency of the knockdown was verified by qRT–PCR (Figure 3C). After the knockdown of METTL3, decreased mRNA expression levels of the osteogenic/odontogenic genes DSPP, DMP1, RUNX2 and ALP were observed (Figure 3D). The expression of osteogenic/odontogenic differentiation-related proteins was detected by Western Blotting, and the data were consistent with the qRT–PCR results (Figure 3G,H). Similarly, as shown in Figure 3I, after the knockdown of METTL3, both ALP activity and mineralized nodules decreased in the siMETTL3 group compared with the control groups (Figure 3I). In contrast, METTL3 overexpression resulted in reverse impacts on the expression levels of these osteogenic/odontogenic differentiation-related genes (Figure 3E–G). The ALP activity and mineralized nodules also increased after the overexpression of METTL3 (Figure 3I).

### 3.4. lncSNHG7 Promoted Osteogenic/Odontogenic Differentiation of hDPSCs

**Figure 4:**
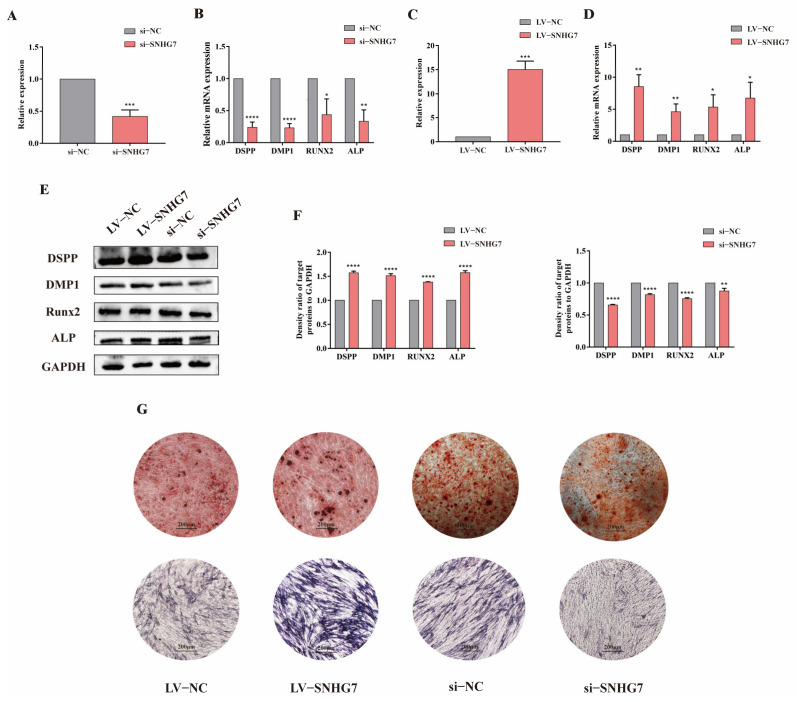
lncSNHG7 promoted osteogenic/odontogenic differentiation of hDPSCs. (**A**,**C**) The knockdown and overexpression effect of si-lncSNHG7 and LV- lncSNHG7 detected by qRT-PCR. (**B**,**D**) mRNA expressions of osteogenic/odontogenic genes detected by qRT-PCR after the knockdown and overexpression of lncSNHG7. (**E**) Western blot results showed the expression level of osteogenic/odontogenic proteins decreased in the si-lncSNHG7 group and increased in the LV- lncSNHG7 group after mineralization. (**F**) The density ratio of target proteins to GAPDH. (**G**) ARS and ALP staining after the knockdown and overexpression of lncSNHG7. The data were represented as means ± SD for each group: * *p* < 0.05, ** *p* < 0.01, *** *p* < 0.001, **** *p* < 0.0001.

The ability of lncSNHG7 to regulate hDPSC osteogenic /odontogenic differentiation was further validated in vitro. siRNA-SNHG7 was constructed and transduced into hDPSCs and was confirmed by qRT–PCR (Figure 4A). As shown in Figure 4B,E,F, lncSNHG7 silencing decreased the mRNA and protein expression levels of DSPP, DMP1, RUNX2 and ALP after induction for 14 days. The ALP activity and mineralized nodules also decreased in the si-SNHG7 group compared with the control group (Figure 4G). However, when lncSNHG7 was overexpressed, the phenotypic changes were reversed (Figure 4C–G). The results above demonstrate that lncSNHG7 is an important regulator that could promote osteogenic/odontogenic differentiation of hDPSCs.

**Figure 5:**
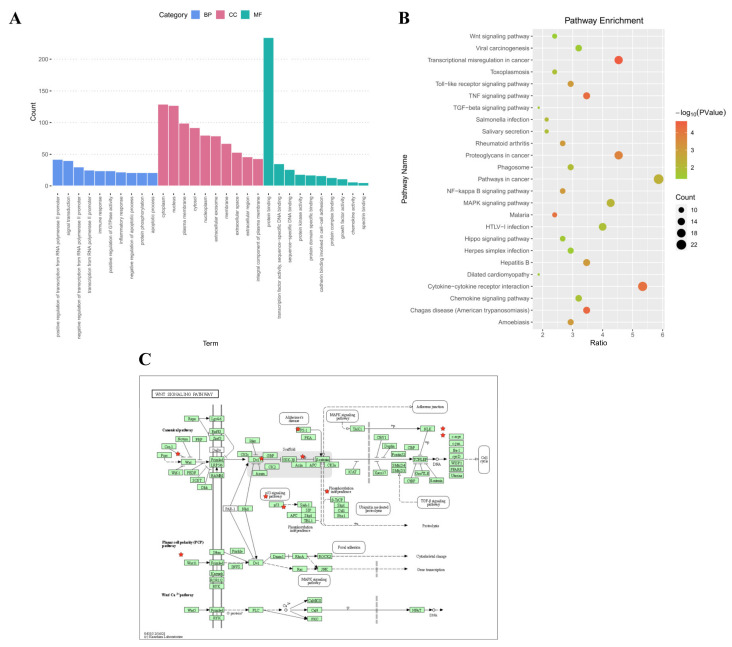
Bioinformatics analysis of lncSNHG7. (**A**,**B**) GO and KEGG pathway analysis of lncSNHG7. (**C**) Wnt signaling pathway map. Red Stars: The potential target gene of lncSNHG7 involved in Wnt signaling pathway.

To further understand the possible roles of lncSNHG7 in functional regulation, GO analysis and KEGG pathway analysis were performed, using the predicted target mRNAs of the lncRNAs based on the StarBase database. The enriched functions in the three GO categories (BP, MF and CC) are shown in Figure 5A. The enriched GO terms for the biological process category are the regulation of transcription from the RNA polymerase II promoter, signal transduction and protein phosphorylation, etc. The molecular-function-structured networks indicate protein binding, transcription factor activity and sequence-specific DNA binding. Through cellular component analysis, the target genes were found to be widely involved in the cytoplasm, nucleus and plasma membrane, etc. The results of the KEGG pathway analysis show that the target mRNAs of lncSNHG7 were enriched in many pathways, including cancer, cytokine–cytokine receptor interactions and transcriptional misregulation in cancer. Four enriched pathways were closely related to osteogenic/odontogenic differentiation, such as MAPK, NF-kappa B, Wnt and TGF-beta (Figure 5B). Figure 5C shows a map of the Wnt signaling pathway.

### 3.5. METTL3 Regulated the m^6^A Modification of lncSNHG7

**Figure 6:**
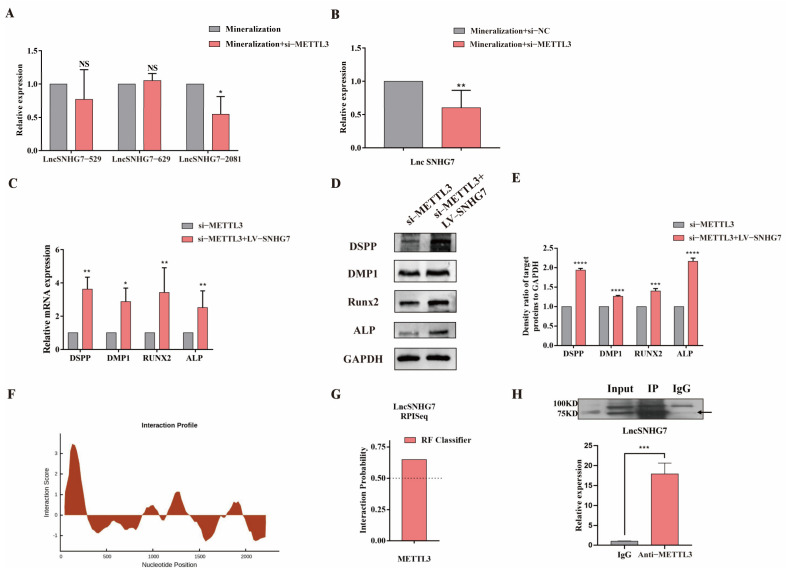
METTL3 regulated the m^6^A modification of lncSNHG7. (**A**) Single base site PCR (MazF) analysis was used to confirm the m^6^A modification site of lncSNHG7 after the knockdown of METTL3. (**B**) The expression level of lncSNHG7 after the knockdown of METTL3. (**C**,**D**) The mRNA and protein expression of osteogenic/ odontogenic genes (DSPP, DMP1, RUNX2 and ALP) under METTL3 knockdown and co-transfection of LV-lncSNHG7. (**E**) The density ratio of target proteins to GAPDH. (**F**) The binding sites between METTL3 and lncSNHG7 were predicted online. (**G**) RPISeq assay showed that METTL3 could bind to lncSNHG7 (The value greater than 0.50 indicates high binding possibility). (**H**) RIP-qPCR confirmed the interaction between METTL3 and lncSNHG7. The data were represented as means ±SD for each group: * *p* < 0.05, ** *p* < 0.01, *** *p* < 0.001, **** *p* < 0.0001. NS: Not Statistically Significant.

METTL3 was shown to be an m^6^A methyltransferase involved in regulating a variety of physiological processes [31]. Because lncSNHG7 has three ACA modification sites with very high confidence, we speculated that METTL3 could target and regulate the m^6^A modification of lncSNHG7. First, after knocking down METTL3, it was found that the m^6^A modification level of lncSNHG7 was reduced (Figure 6A), and the expression level of lncSNHG7 was also reduced, indicating that METTL3 not only regulated the m^6^A modification of lncSNHG7, but also affected its expression (Figure 6B). The overexpression of lncSNHG7 could rescue the reduced expression levels of DSPP, DMP1, RUNX2 and ALP caused by the knockdown of METTL3 (Figure 6C–E). In addition, we analyzed the nucleotide sequence of lncSNHG7 by catRAPID and found that nucleotides at 70–121 and 495–546 had a high potential to bind with proteins (Figure 6F). The possibility of the combination of lncSNHG7 and METTL3 is shown in Figure 6G and the binding between METTL3 and lncSNHG7 was confirmed by RIP-qPCR (Figure 6H).

### 3.6. The METTL3/lncSNHG7 Axis Regulated the Wnt/β-Catenin Signaling Pathway

**Figure 7:**
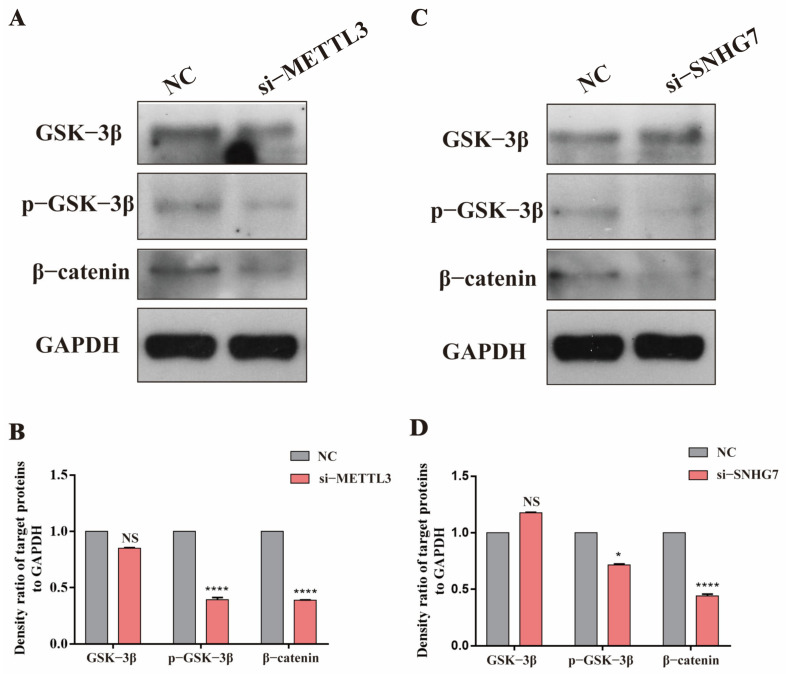
Demonstration of the METTL3/lncSNHG7axis and its regulatory analysis of Wnt/β-catenin signaling pathway. (**A**,**C**) The protein expression levels of β-catenin and GSK-3βphosphorylation under lncSNHG7 and METTL3 knockdown. (**B**,**D**) The density ratio of target proteins to GAPDH. The data were represented as means ± SD for each group: * *p* < 0.05, **** *p* < 0.0001, NS: Not Statistically Significant.

Bioinformatics analysis predicted that the lncSNHG7 target gene was enriched in the Wnt/β-catenin signaling pathway. We speculated that METTL3 could affect the Wnt/β-catenin signaling pathway by regulating the m^6^A modification of lncSNHG7 to ultimately promote the osteogenic/odontogenic differentiation of hDPSCs. First, lncSNHG7 knockdown resulted in decreased phosphorylation of the key protein GSK-3β in the Wnt/β-catenin signaling pathway and the expression of β-catenin also decreased (Figure 7A,B), indicating that lncSNHG7 activated the Wnt/β-catenin signaling pathway. After METTL3 was knocked down, a decreased expression of phosphorylation of GSK-3β and β-catenin were then observed (Figure 7C,D). These results confirm the presence of the METTL3/lncSNHG7 axis, which could regulate the Wnt/β-catenin signaling pathway and affect the osteogenic/odontogenic differentiation of hDPSCs.

## 4. Discussion

The m^6^A modification is the most common modification in post-transcriptional RNA. It can also regulate noncoding RNAs, such as miRNAs, lncRNAs and circRNAs. The change in its level may be closely related to the metabolism and function of RNA. It has been reported that m^6^A modification is involved in the biological processes of a variety of stem cells and plays an important role in bone metabolism. For example, the demethylase ALKBH5 can promote the expression of osteogenic genes [32]. METTL14 plays a regulatory role in osteoporosis. METTL14 can promote osteoclast activity by inhibiting miRNA expression [33]. The importance of METTL3-mediated m^6^A methylation of XIST in OPLL provides new insights into therapeutic strategies for OPLL [34]. However, few studies have been conducted to examine m^6^A modification of hDPSCs. Luo et al. demonstrated that METTL3 plays a regulatory role in the cell cycle [17]. In addition, METTL3 was found to be involved in the development of tooth roots, the deletion of which led to the reduction in odontogenic differentiation, shortening of molar roots and thinning of dentin by weakening the translation efficiency of nuclear factor IC (NFIC) (a key regulator of tooth roots) [35]. METTL3 can also directly interact with ATP citrate lyase (ACLY) and mitochondrial citrate transporter (SLC25A1) to then further affect the glycolysis pathway and glucose metabolism during the osteogenesis of hDPSCs [36]. However, based on the literature, the mechanism underlying the m^6^A modification involved in bone metabolism of hDPSCs has not been fully clarified. It is still controversial and requires further exploration. Recent studies have shown that m^6^A modification can also affect the stability and metabolism of lncRNAs [37,38,39]. However, to the best of our knowledge, there has been no research on the regulation of bone homeostasis and bone tissue engineering by m^6^A of lncRNA in hDPSCs. Therefore, this study is expected to provide a new theoretical basis for the study of the mechanism of osteogenic/odontogenic differentiation in hDPSCs.

In this study, we first identified the m^6^A modification of lncSNHG7 in hDPSCs by a MazF experiment. Its occurrence region was the 2081 site of the conserved motif region containing the core ACA sequence, but whether the m^6^A modification of lncSNHG7 still occurs at other sites requires further study. The potential regulatory mechanism of METTL3 in the osteogenic/odontogenic differentiation of hDPSCs was then discussed. Knockdown and overexpression of METTL3, respectively, reduced and increased expression levels of DSPP, DMP1, RUNX2 and ALP, ALP activity and the level of mineralized nodules, which supported the positive regulatory role of METTL3 in the mineralization of hDPSCs, consistent with other studies [40,41]. However, other studies have revealed that METTL3 can inhibit osteogenesis through m^6^A modification [42,43]. Altogether, these results confirm that METTL3 may be an important regulator of osteogenic/odontogenic differentiation, but the specific regulation of different stem cells requires further study.

lncRNAs can regulate gene expression at the level of chromatin modification, transcription and post-transcriptional processing, and are very important in almost all biological processes, including pluripotency, cell development, the immune response and differentiation. Many studies have shown that lncRNAs play an important role in the osteogenic/odontogenic differentiation of hDPSCs [29,44]. However, there has been no research on the regulation of lncRNA m^6^A modification in hDPSC osteogenic/odontogenic differentiation. In our study, METTL3 increased m^6^A methylation and the expression levels of lncSNHG7, leading to promotion of the osteogenic/odontogenic differentiation of hDPSCs. These findings reveal a new role of METTL3 in hDPSCs, showing that METTL3 could promote osteogenic/odontogenic differentiation of hDPSCs through the upregulation of lncSNHG7.

Osteogenic/odontogenic differentiation is regulated by a variety of signaling pathways, including the Wnt/β-catenin signaling pathway. The expression of β-catenin is very important for tooth formation, and β-catenin may play an important role in BMP-9-induced osteogenic and odontogenic signal transduction [45]. Recent data suggested that the treated dentin matrix directly targeted GSK-3β and activated the typical Wnt/β-catenin signaling pathway to promote odontogenic differentiation of hDPSCs [46]. These reports strongly suggested that Wnt/β-catenin signaling regulated osteogenic/odontogenic differentiation. In the present study, through bioinformatics analysis of lncSNHG7, we found that osteogenic/odontogenic differentiation was enriched in the Wnt/β-catenin signaling pathway. We, therefore, speculate that METTL3 can affect the Wnt/β-catenin signaling pathway and that it ultimately promoted the osteogenic/odontogenic differentiation of hDPSCs by regulating the m^6^A modification of lncSNHG7. Our results show that knockdown of lncSNHG7 and METTL3 led to decreased expression levels of p-GSK-3β and β-catenin in the Wnt/β-catenin signaling pathway.

Improving the differentiation ability of stem cells before clinical application is necessary. Studies demonstrated that Alx3-Wnt3a promoted angiogenesis and newly formed dentin with a structural–mechanical equivalency suggesting that conceptualizing recombinant human Wnt3a delivery in disinfected root canals is one of the possible methods in tooth regeneration [47]. The present study revealed that METTL3-mediated lncSNHG7 m^6^A modification is involved in the Wnt/β-catenin signaling pathway and could promote osteogenic/odontogenic differentiation of hDPSCs. Similarly, hDPSCs stably transfected with METTL3-lncSNHG7 and synthetic scaffolds (such as collagen, hydrogel, decellularized bioscaffold and nanofibrous spongy microspheres) might be constructed and injected into the disinfected root canal to replace the original root-filling materials for regeneration. Until then, however, the specific underlying mechanisms on the correlation of m^6^A modification, lncSNHG7 and the Wnt/β-catenin signaling pathway in the process of osteogenic/odontogenic differentiation require further exploration and will be a focus of our future studies.

## 5. Conclusions

**Figure 8:**
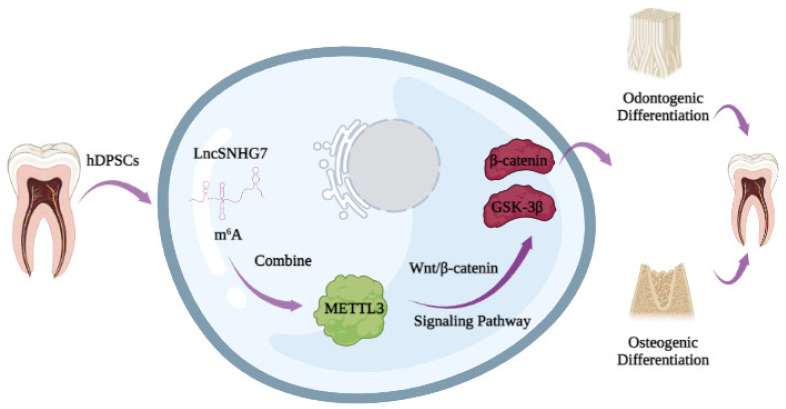
A schematic illustration of the molecular mechanism of lncSNHG7 promoting osteogenic/odontogenic differentiation of hDPSCs. This schematic illustration created in BioRender.com (Accessed on 20 September 2022).

To conclude, the present study revealed that lncSNHG7, mediated by METTL3 through m^6^A modification, could activate the Wnt/β-catenin signaling pathway to promote the osteogenic/odontogenic differentiation of hDPSCs (Figure 8). As the activation of molecular pathways is a pivotal characteristic in tissue regeneration, our study might provide a key clue for the future application of hDPSCs in clinically regenerative endodontic treatment. The function of METTL3-mediated lncSNHG7 in vivo will be investigated in our future studies.

## Figures and Tables

**Table 1:** The sequences of the primers used in PCR.

Gene	Sequence 5′–3′	
GAPDH	Forward:	TCAACAGCGACACCCACTC
	Reverse:	GCTGTAGCCAAATTCGTTGTC
ALP	Forward:	CCAAAGGCTTCTTCTTGCTG
	Reverse:	CCACCAAATGTGAAGACGTG
Runx2	Forward:	TCGCCAGGCTTCATAGCAAA
	Reverse:	GGCCTTGGGTAAGGCAGATT
DSPP	Forward:	CAGCAGCGACAGCAGTGATAGC
	Reverse:	TGTCACTGTCACTGTCACTTCCATTG
DMP1	Forward:	CTCCGAGTTGGACGATGAGG
	Reverse:	TCATGCCTGCACTGTTCATTC
METTL3	Forward:	GAGGAGTGCATGAAAGCCAG
	Reverse:	GGCCTCAGAATCCATGCAAG
METTL14	Forward:	GACGGGGACTTCATTCATGC
	Reverse:	CCAGCCTGGTCGAATTGTAC
IGF2BP1	Forward:	TGAAGCTGGAGACCCACATA
	Reverse:	GGGTCTGGTCTCTTGGTACT
IGF2BP2	Forward:	AGTGGAATTGCATGGGAAAATCA
	Reverse:	CAACGGCGGTTTCTGTGTC
IGF2BP3	Forward:	TATATCGGAAACCTCAGCGAGA
	Reverse:	GGACCGAGTGCTCAACTTCT
ALKBH5	Forward:	ACCCCATCCACATCTTCGAG
	Reverse:	CTTGATGTCCTGAGGCCGTA
HNRNPA2B1	Forward:	CAGTTCTCACTACAGCGCCA
	Reverse:	TTCCTCTCCAAAGGAACAGTTT
FTO	Forward:	AGACACCTGGTTTGGCGATA
	Reverse:	CCAAGGTTCCTGTTGAGCAC
YTHDC1	Forward:	CTTCTGATGAGCAAGGGAACAA
	Reverse:	GGCCTCACTTCGAGTGTCATAA
YTHDF1	Forward:	ACCTGTCCAGCTATTACCCG
	Reverse:	TGGTGAGGTATGGAATCGGAG
FMR1	Forward:	TATGCAGCATGTGATGCAACT
	Reverse:	TTGTGGCAGGTTTGTTGGGAT
HNRNPC	Forward:	GTTACCAACAAGACAGATCCTCG
	Reverse:	AGGCAAAGCCCTTATGAACAG
WTAP	Forward:	ACGCAGGGAGAACATTCTTG
	Reverse:	CACACTCGGCTGCTGAACT
lncSNHG7	Forward:	TTGCTGGCGTCTCGGTTAAT
	Reverse:	GGAAGTCCATCACAGGCGAA

## Data Availability

Not applicable.

## Supplementary Materials

The following supporting information can be downloaded at: https://www.mdpi.com/article/10.3390/jcm12010113/s1, Table S1. Prediction of m6A modification sites of lncSNHG7. Table S2 Prediction of m6A modifying enzymes possibly bound to lncSNHG7.

## Abbreviations

(hDPSCs) Human dental pulp stem cells, (m^6^A) N6 methyladenosine, (METTL3) methyltransferase 3, (lncRNAs) Long noncoding RNAs, (ncRNA) oncoding RNA, (miRNA) microRNA, (DMEM) Dulbecco’s modified Eagle’s medium, (FBS) Fetal bovine serum, (qRT-PCR) real-time quantitative polymerase chain reaction, (ALP) Alkaline Phosphatase, (ARS) Alizarin Red Staining, (GAPDH) glyceraldehyde-3-phosphate dehydrogenase, (siRNAs) small interfering RNAs, (GO) Gene Ontology, (KEGG) Kyoto Encyclopedia of Genes and Genomes, (BP) biological processes, (CC) cellular components, (MF) molecular functions, (RIP) RNA-binding protein immunoprecipitation, (DSPP) dentin sialophosphoprotein, (DMP1) dentin matrix acidic phosphoprotein 1, (Runx2) runt-related transcription factor 2.
