# Supplementary material for: METTL3-Mediated lncSNHG7 m6A Modification in the Osteogenic/Odontogenic Differentiation of Human Dental Stem Cells
Source: J Clin Med. 2022 Dec 23;12(1):113. doi: 10.3390/jcm12010113 (PMC9821659; doi:10.3390/jcm12010113)
Supplement: Supplementary file 1 [file jcm-12-00113-s001.zip › Supplementary Table S1.pdf]

Table S1 Prediction of m^6A modification sites of lncSNHG7

Number	Position	Sequence context	Score (binary)	Score (knn)	Score (spectrum)	Score (combined)	Decision
1	393	GAGGU GACUU CGCCU<br>GUGAU GGACU UCCAG<br>UGUGA GCACU GGCCA	0.767	0.792	0.498	0.66	(High confidence)
2	529	GAGUA ACAAA CCCCC<br>UUGGA GGACU CUCCU<br>GCCGG GAUGU CCAUG	0.702	0.704	0.688	0.697	(Very high confidence)
3	614	UGGAA UCCAG AGCCC<br>UGACG GGACA GCAGC<br>AGCAG GAACU CGUUA	0.602	0.632	0.674	0.632	(High confidence)
4	629	UGACG GGACA GCAGC<br>AGCAG GAACU CGUUA<br>CGCUG CAGCA GGGCC	0.677	0.682	0.673	0.676	(Very high confidence)
5	659	CGCUG CAGCA GGGCC<br>CACAC GGACU UGGCU<br>UCCGC CAGGG ACACC	0.746	0.831	0.549	0.671	(High confidence)
6	677	ACGGA CUUGG CUUCC<br>GCCAG GGACA CCCGG<br>UUGUC CUUGU UGAAG	0.618	0.801	0.578	0.611	(High confidence)
7	715	GUUGA AGUCA GCCAU<br>GAGCA GGACC UGGCC<br>AACCA GCGCC GGCAG	0.602	0.794	0.667	0.637	(High confidence)
8	1163	UUCGG GGCAC CUGCC<br>CUGCU GGACA CACGG	0.627	0.674	0.628	0.63	(High confidence)

9	1177	CCCUU GACUG CAGGA CCUGC UGGAC ACACG GCCCU UGACU GCAGG AGCCU CCCCU CGACC	0.554	0.743	0.615	0.588	(Moderate confidence)
10	1388	UGGGC AUAUG ACCGU GGUGU GGACU CCUGC CCUGC CCCCU AGGAG	0.682	0.761	0.329	0.544	(Low confidence)
11	1603	CCAGA GCCCG CUGUA CACCU GGACA GGGCC ACAGA GGUCC UCCGU	0.672	0.804	0.631	0.662	(High confidence)
12	1630	GCCAC AGAGG UCCUC CGUGC AGACA UCCCC CUCCC ACUGC ACGGG	0.476	0.813	0.648	0.562	(Moderate confidence)
13	2081	CACAG CAAAG GCCAC UGCCU GGACA UGAGA CACAC ACCAC ACCCA	0.695	0.749	0.735	0.714	(Very high confidence)
14	2088	AAGGC CACUG CCUGG ACAUG AGACA CACAC CACAC CCAGU GUCGA	0.46	0.649	0.731	0.578	(Moderate confidence)
15	2136	CACGC CAGGG CCAGA GGCAG GAACC UGGAG GCAGC UCUCC GCCCA	0.437	0.534	0.763	0.572	(Moderate confidence)

16	2175	CGCCC AGCCG ACCCA GCUCU GGACC AUCCA GGCAU UGGCC GGUGA	0.583	0.622	0.765	0.658	(High confidence)
17	2198	CCAUC CAGGC AUUGG CCGGU GAACU AGAAU UCACA CUAGU CCCUA	0.548	0.493	0.718	0.613	(High confidence)
18	2262	CCACA CGCGC GCUCU CUGCC UGACU CUUCA UUCCU GCCUC GGGUG	0.4 85	0.642	0.685	0.573	(Moderate confidence)
19	2349	CCUGC CCGGA AUAGU AAGUG AGACA UUUCU G---- ----- -----	0.5 12	0.614	0.561	0.537	(Low confidence)
