# Supplementary material for: METTL3-Mediated lncSNHG7 m6A Modification in the Osteogenic/Odontogenic Differentiation of Human Dental Stem Cells
Source: J Clin Med. 2022 Dec 23;12(1):113. doi: 10.3390/jcm12010113 (PMC9821659; doi:10.3390/jcm12010113)
Supplement: Supplementary file 1 [file jcm-12-00113-s001.zip › Supplementary Table S2.pdf]

Table S2 Prediction of m6A modifying enzymes possibly bound to lncSNHG7

RBP	GeneID	GeneName	GeneType	ClusterNum	ClipExpNum	ClipSiteNum	HepG2(log2FC)	K562(log2FC)	Pan-Cancer
IGF2BP1	ENSG00000233016	SNHG7	antisense	15	5	25	-0.649	NA	12
IGF2BP2	ENSG00000233016	SNHG7	antisense	20	8	42	-0.753	NA	14
IGF2BP3	ENSG00000233016	SNHG7	antisense	13	9	24	-1.224	NA	17
FTO	ENSG00000233016	SNHG7	antisense	5	1	5	NA	NA	21
FMR1	ENSG00000233016	SNHG7	antisense	17	8	44	NA	NA	19
WTAP	ENSG00000233016	SNHG7	antisense	3	2	4	NA	NA	13
YTHDC1	ENSG00000233016	SNHG7	antisense	6	5	9	NA	NA	12
YTHDF1	ENSG00000233016	SNHG7	antisense	7	2	7	NA	NA	16
METTL3	ENSG00000233016	SNHG7	antisense	1	4	4	NA	NA	20
METTL14	ENSG00000233016	SNHG7	antisense	4	4	9	NA	NA	17
ALKBH5	ENSG00000233016	SNHG7	antisense	1	1	1	NA	NA	12
HNRNPA2B1	ENSG00000233016	SNHG7	antisense	3	6	13	NA	0.696	13
HNRNPC	ENSG00000233016	SNHG7	antisense	17	8	39	NA	NA	16
